# A review of split liver transplantation with full right/left hemi-liver grafts for 2 adult recipients

**DOI:** 10.1097/MD.0000000000027369

**Published:** 2021-10-01

**Authors:** Kun-Ming Chan, Hao-Chien Hung, Jin-Chiao Lee, Tsung-Han Wu, Yu-Chao Wang, Chih-Hsien Cheng, Chen-Fang Lee, Ting-Jung Wu, Hong-Shiue Chou, Wei-Chen Lee

**Affiliations:** Department of General Surgery and Chang Gung Transplantation Institute, Chang Gung Memorial Hospital at Linkou, Chang Gung University College of Medicine, Taoyuan, Taiwan.

**Keywords:** adult recipient, deceased donor, donor and recipient matching, hemi-liver graft, outcomes, split liver transplantation

## Abstract

Liver transplantation has become a routine operation in many transplantation centers worldwide. However, liver graft availability fails to meet patient demands. Split liver transplantation (SPLT), which divides a deceased donor liver into 2 partial liver grafts, is a promising strategy for increasing graft availability for transplantation and ameliorating organ shortage to a certain degree. However, the transplantation community has not yet reached a consensus on SPLT because of the variable results. Specifically, SPLT for 2 adult recipients using full right/left hemi-liver grafts is clinically more challenging in terms of surgical technique and potential postoperative complications. Therefore, this review summarizes the current status of SPLT, focusing on the transplantation of adult recipients. Furthermore, the initiation of the SPLT program, donor allocation, surgical aspects, recipient outcomes, and obstacles to developing this procedure will be thoroughly discussed. This information might help provide an optimal strategy for implementing SPLT for 2 adult recipients among current transplantation societies. Meanwhile, potential obstacles to SPLT might be overcome in the near future with growing knowledge, experience, and refinement of surgical techniques. Ultimately, the widespread diffusion of SPLT may increase graft availability and mitigate organ donation shortages.

## 1 Introduction

Liver transplantation (LT) is common solid organ transplantation that is routinely performed in many transplantation centers worldwide. However, the available liver grafts fail to fulfill the needs of the number of patients requiring LT. Advances in surgical techniques and medical facilities have evolved to maximize liver grafts for transplantation, including living donor LT (LDLT), blood type-incompatible LT, donor from circulation death LT, and deceased donor split LT (SPLT).^[1]^ Of these, SPLT was the earliest attempted modality but remains underutilized as an alternative to increasing graft for LT.^[2,3]^ Conventionally, the strategy of splitting a liver graft from a deceased donor was initially performed to offer a smaller graft for pediatric patients and a larger right graft for adults,^[3]^ and it remains the most accepted form of SPLT nowadays. However, SPLT, which divides a deceased donor liver into 2 full hemi-liver grafts for 2 adult recipients, has not achieved consensus among the transplantation community because of variable results.^[4–6]^ Accordingly, SPLT for 2 adult recipients has not yet gained acceptance as a common practice.

As such, SPLT of 2 full hemi-liver grafts for 2 adult recipients remains technically more challenging during organ procurement than other types of deceased donor LT. Meanwhile, potential complications could also be a drawback of utilizing SPLT for 2 adult recipients.^[7–12]^ Therefore, this review summarizes the current status of SPLT, focusing on transplantation for adult recipients. Specifically, the process of SPLT, including the initiation of the transplantation program, donor-recipient matching, surgical aspects, outcomes, and obstacles to be solved for developing this procedure will be thoroughly discussed. This information might help provide an optimal paradigm for the implementation of SPLT for 2 adult recipients under the current allocation system of the transplantation society.

## 2 Initiation of the SPLT program

Transection of the hepatic parenchyma has evolved over the last several decades and has contributed dramatically to the safety and effectiveness of liver resection. Specifically, the introduction of ultrasonic cavitation devices using low-frequency ultrasound energy to dissect or fragment hepatic tissues was undoubtedly a transition to a new era of safe liver resection. This surgical device has the advantages of less blood loss and improved visibility of intrahepatic vascular pedicles, and has increased the success rate and safety of liver resection.

Based on the confidence gained from the experience of liver resection, reduced-size LT in children using a partial liver graft from a deceased donor was first attempted by Bismuth and Houssin in 1984.^[13]^ Subsequently, Pichlmayr et al reported the first SPLT implanting a smaller left lateral graft into a child and a larger right graft into adults in 1988.^[3]^ Soon after, Bismuth et al described the possibility of dividing a deceased donor liver into 2 full hemi-liver grafts for 2 adult recipients.^[14]^ Although both recipients died after SPLT, this attempt opened a new window for LT. Meanwhile, Strong et al reported another milestone in 1990 with LT using a portion of the liver from a living donor.^[15]^ Moreover, LDLT further evolved from using the left lateral graft for children to use a right lobe graft in an adult recipient. As a result of these great advances in the field of LDLT, it has now become a common and routine operation in many major LT centers worldwide.^[16]^

However, SPLT for 2 adults has been developed from the stepwise progression of liver resection, LT, and LDLT. With this approach, a whole deceased donor liver is divided into 2 functioning hemi-liver allografts. Although this might increase the number of donor organs, it remains highly challenging in terms of surgical techniques. Based on an analysis from the United Network for Organ Sharing of the United States, nearly half of the SPLTs were concentrated in 10 transplantation centers that performed ≥20 SPLTs during the study period.^[2]^ Of these, only less than 10% were SPLT for 2 adult recipients. As such, LT volume might be an important factor attributed to the infrequent utilization of SPLT. Therefore, the prerequisite for the initiation of the SPLT program might be fulfilled by an adequate volume and experience in liver resection and LT. Moreover, a better understanding and experience in LDLT for adults might be pivotal for the implementation of SPLT for 2 adult recipients. In addition, the improvement of allocation policies for better patient and donor selection, technical refinements, and encouraging widespread diffusion of the surgical technique are all factors related to the success of SPLT.

## 3 Donor

### 3.1 Donor selection

In general, careful donor selection is essential for successful SPLT. However, there is currently no formal algorithm for decision-making while splitting liver grafts from deceased donors. The vital conditions of the donor and graft quality are both important for determining whether the liver graft is suitable for splitting, and splitting for 2 full hemi-liver grafts should be more rigorous than splitting a smaller graft for pediatric recipients. Based on the reviewed data, there are few general principles related to donor criteria proposed for the consideration of split liver grafts for LT (Table 1). Briefly, the donor should be hemodynamically stable, with well-preserved liver function, with only mild fatty changes in the liver parenchyma, and aged between 15 and 55 years.^[11,17–20]^

**Table 1:** General principles of donor selection for splitting liver grafts into two full hemi-liver grafts.

Age 15–55 yrs
Hemodynamics stable, and no cardiac arrest episodes
Minimal inotropic agent
No macroscopic evidence of hepatic steatosis
No obvious systemic bacterial infection
No prolong intensive care unit stay, better less than 5 days
liver transaminase within 5 folds or less
Serum sodium <160 mg/dL

Additionally, Doppler sonography of the liver prior to organ recovery might be helpful in identifying hepatic steatosis and vascular distribution of the liver graft. Specifically, the expected liver volume of hemi-liver grafts can be estimated by calculating the standard liver volume and using the equation utilizing the maximum diameter of the portal vein as described by Lee et al^[21,22]^ Nonetheless, the utilization of computed tomography to measure the liver volume and anatomical variations of deceased donors might be helpful, but risks to donors during the examination and ethical issues should also be considered.

### 3.2 Surgical techniques for liver split

During organ recovery, a thorough inspection of the liver to evaluate the sizes of the right and left lobes, vascular anatomy, and consistency of the hepatic parenchyma by donor surgeons is essential. Cholangiography for the assessment of biliary tract distribution is also mandatory for determining the cut point of the left and right hepatic ducts. Separation of hepatic parenchyma could be performed ex vivo and in situ depending on the hemodynamic stability of donors or as per the surgeon's preference, as previously described.^[10,20,23–26]^ Nonetheless, the timeframe of hepatic transection should be cautiously considered and minimized as much as possible in both approaches, which could prevent prolonged cold ischemia time in grafts from ex vivo splits and hemodynamic instability in donors during in situ splits. Generally, in situ splits are preferred by transplantation centers with extensive experience in LDLT for hemodynamically stable donors.^[18,20,27,28]^ Moreover, the cut surface of the hemi-grafts could be sealed with fibrin glue, which might help to reduce bleeding after graft reperfusion.^[29]^

However, the most important challenge facing liver graft splitting is the determination of major vessels and bile duct sharing, an aspect that still suffers from a lack of consensus. Despite this, the principal concept is to divide the major vessels and the bile duct at an optimal point, making it easy to reconstruct vascular inflow and outflow as well as bile duct drainage for graft implantation. Thus, it can prevent the risk of surgical complications from multiple complex anastomoses.

**Figure 1:**
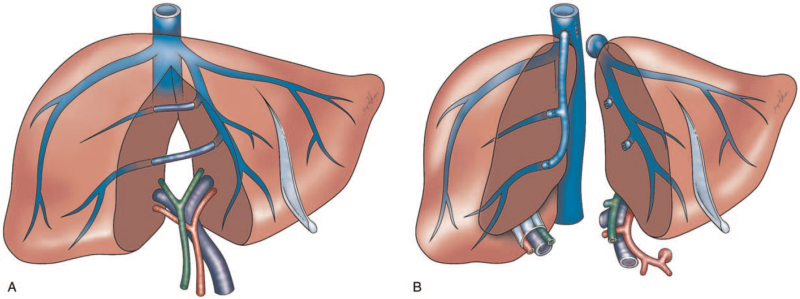
Illustration of the most common form of liver splitting for full hemi-liver grafts. (A) The transection line is performed along the Cantlie line at the right side of MHV. (B) The inferior vena cava is preserved for the right hemi-liver graft. The main hepatic arterial trunk, main portal vein, and the common bile duct are retained with the left hemi-liver graft during back-table preparation. The left hepatic vein and middle hepatic vein of the left hemi-liver graft are prepared as a common orifice for outflow reconstruction of graft implantation. The venous tributaries of MHV (V5 and V8) in the right hemi-liver graft should be reconstructed as appropriate. MHV = middle hepatic vein.

**Figure 2:**
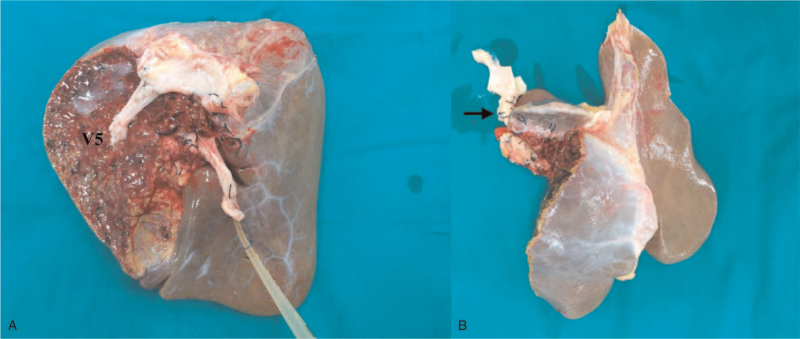
Split hemi-liver grafts. (A) The right hemi-liver graft. The venous tributaries of the segment 5 (V5) and 8 (V8) were reconstructed by venous graft. (B) The left-hemi-liver graft. Arrow indicates the celiac trunk of the graft. The cut surface of the hepatic parenchyma could be sealed with fibrin glue that could help to reduce bleeding after graft reperfusion.

The most common form of sharing vascular and biliary structure for splitting liver graft is illustrated in Figure 1. Usually, the transection line of liver parenchyma is similar to the LDLT using a right liver graft without a middle hepatic vein (MHV). The inferior vena cava (IVC) is preserved for the right hemi-liver graft. Then, the left hepatic vein and middle hepatic vein are prepared as a common orifice during back-table graft preparation. Additionally, the venous tributaries of the MHV should be reconstructed for the right hemi-liver graft as appropriate. (Fig. 2) Generally, the necessity for the reconstruction of MHV tributaries, mainly the drainage of segments 5 (V5) and 8 (V8), could also be determined based on numerous proposed algorithms for right liver graft LT.^[30–33]^ With growing experience in LT as well as LDLT, the implementation of microscopic reconstruction for small hepatic arteries and bile ducts may also be accomplished without much difficulty in SPLT using hemi-liver grafts from deceased donors.^[34–36]^

### 3.3 Machine perfusion

Machine perfusion has emerged as a novel technique for extending the utilization of marginal liver grafts from non-heart-beating donors.^[37]^ Additionally, the splitting of livers has also been attempted in this context. However, the majority of reports were preclinical or animal models, in which split liver grafts were not transplanted.^[38–42]^ More recently, ex vivo liver splitting during dynamic machine perfusion has been well described, in which a left lateral liver graft and an extended right liver graft were successfully transplanted into 2 recipients.^[43,44]^ Importantly, experience from these early attempts perhaps could encourage and facilitate the use of machine perfusion for split full hemi-liver grafts for adult recipients in this way. Nonetheless, more studies are warranted to evaluate the potential clinical benefits of machine perfusion in SPLT and to bring this strategy to clinical application in the future.

## 4 Recipient

### 4.1 Recipient matching

Currently, the whole transplantation society fully respects the priority indications for LT based on the algorithm from the United Network for Organ Sharing (UNOS), the model for end-stage liver disease (MELD) score, and the time on the waiting list. Accordingly, donors eligible for split liver grafts should also rely on recipient matching from the waiting list. Under this algorithm of allocation, liver grafts are usually directed to the sickest recipients with UNOS status I or the highest MELD score.

However, a number of concerns must be taken into consideration when splitting liver grafts for transplantation. First, size matching in terms of graft-to-recipient weight ratio (GRWR) is of utmost importance for partial liver graft LT. Although a minimum GRWR of 0.6% could be acceptable for LDLT, an estimated GRWR of at least 0.8% is recommended for split liver grafts from deceased donors for adult recipients.^[19,20,45,46]^ A potential liver graft should be transplanted as a whole liver if an optimal GRWR cannot be offered to the first-priority recipient. Second, decreased survival has been observed with split liver grafts in high-risk recipients^[17,47,48]^; thus, surgeons might be reluctant to use split hemi-liver grafts in patients with a high MELD score. Indeed, the sicker recipient might theoretically need a greater liver mass to compensate for the illness during the postoperative recovery period. Nonetheless, numerous reports have shown that the results of SPLT for adult recipients, even at higher MELD scores (ie, >35), were more than acceptable and comparable to those of whole LT.^[19,49–51]^ Therefore, SPLT for adult recipients with high-MELD scores is justified and could be broadly applied to this candidate population.

Moreover, leftover hemi-liver graft sharing is another significant issue for the utilization of SPLT in adult recipients. The fear that a good-quality graft divided into 2 marginal hemi-liver grafts might endanger the recipients has withdrawn most transplantation centers from using the leftover hemi-liver grafts obtained from SPLT for adult recipients. Based on an analysis of the UNOS registration data, 16% of the secondary split grafts were discarded for various reasons.^[2]^ In such circumstances, the initial intent to expand the donor pool for LT no longer exists. Therefore, the collaboration between different centers with adequate experience in LT and/or SPLT is essential for successfully sharing hemi-liver grafts. Currently, there is no formal allocation system for sharing hemi-liver grafts from SPLT in the transplantation community.^[2,18,29]^ The secondary hemi-liver graft is usually allocated to a size-matched recipient on the wait-list of the same transplantation centers.

However, the Split-Liver Group of the North Italy Transplantation program has developed a promising SPLT policy for 2 adult recipients.^[17]^ All adult recipients on the waiting list for LT could be selected at any time of organ allocation for SPLT based on the blood-type compatibility and donor-recipient size match. The Split-Liver Network program can make real-time matches between the potential donor and registered patients according to size-based matched donor/patients, and determine hemi-liver graft allocation once the donor is considered eligible for the split liver procedure. All transplantation centers in this program could share hemi-liver grafts for SPLT. Accordingly, widely encouraging SPLT might be able to mitigate the current severe organ shortage and reduce wait-list mortality.

### 4.2 Recipient outcomes

The majority of results gained from SPLT for 2 adult recipients remain variable and have only been reported by a few experienced centers. Table 2 summarizes a number of reported series that had performed more than 20 adult SPLTs. Outcomes generated from a few SPLT centers showed that 5-year patient survival rates (63%–69%) were inferior to those of whole LT in adult recipients.^[17,52–54]^ Whereas numerous other reports demonstrated that SPLT, LDLT, and whole LT had comparable outcomes.^[18–20,55–59]^ However, one would expect that outcomes of SPLT for adult recipients might have progressively improved over time owing to the growing knowledge and experience with LT.

**Table 2:** Reported series with more than 20 adult split liver transplantations.

Author, Year, country	Patient numbers	Major concerns and comments	Patient survival
Kong et al, 2020, China^[63]^	47 (L: 27, R:20)	SPLT had more Clavien–Dindo grade III–V complications, longer hospitalization duration, and higher mortality within 45 days	80% (5 yrs)
Chan et al, 2019, Taiwan^[18]^	100 (L:48, R:52)	A graft weight more than 580 g had a significantly better outcome.	59% (5 yrs)
Herden et al, 2018, Germany^[54]^	44 (27 adults, 17 pediatrics)	SPLT remains a rare procedure restricted to experienced liver transplant centers.	L:91% (10 yrs) R:57% (10 yrs)
Halac et al, 2016, Argentina (multi-centers)^[62]^	111 (L:57, R:54)	1. Biliary complications were the most frequent complications. 2. Adequate donor selection and reducing cold ischemia time are crucial for optimizing results.	L: 83% (3 yrs) R:78% (3 yrs)
Zimmerman et al, 2016, USA (UNOS database)^[57]^	768 (L:117, R:651)	1. There was no difference in allograft or patient survival associated with the graft types after liver transplantation. 2. SPLT is a valuable and safe option to expand the donor pool.	L:67% (5 yrs) R:74% (5 yrs)
Aseni, 2014, Italy^[17]^	64 (L:32, R:32)	Donor age, female recipient, recipient body weight, and HCV cirrhosis negatively affected patients’ survival.	65% (5 yrs)
Hashimoto et al, 2014, USA^[19]^	25 (L:10, R:32)	SPLT can achieve excellent outcomes under the MELD allocation, but the routine application is still controversial due to various challenges.	88% (5)
Lee et al, 2013, Taiwan^[20]^	42 (R:21, L:21)	The GRWR was better more than 1% to avoid early mortality.	69% (5 yrs)
Cescon et al, 2009, Italy^[59]^	22 (L:9, R:13)	Post-operative hyperbilirubinemia were significantly higher in recipients of left-liver grafts versus right-liver grafts.	90%
Humar et al, 2008, USA^[51]^	31 (L:15, R:16)	The status of the recipient is probably a more important determinant of outcome than graft type or donor source.	74% (3 yrs)
Broering et al, 2005, Germany^[4]^	35 (L:19, R:16)	The key to success is the choice of adequate deceased donors and recipients.	R:87% (1 yr) L:89% (1 yr)
Zambelli et al, 2002, Italy (5 centers)^[56]^	43 (R:21, L:22)	1. Hospital mortality was 23% with sepsis as the main cause. 2. Multicenter collaboration in sharing of grafts is feasible and can help to face the organizational limits, thus increasing the diffusion of full-right-full-left SPLT.	63% (10 yrs)
Azoulay et al, 2001, France^[10]^	34 (R: 17, L: 17)	Graft steatosis, recipient's condition, and hospital stay were associated with graft failure.	R: 74% (2 yrs) L: 64% (2 yrs)

GRWR = graft-to-recipient weight ratio, L = left, R = right, SPLT = split liver transplantation.

In addition, factors affecting patient and graft survival rates were not definite as well. Numerous risk factors have been observed for poor outcomes of SPLT for adult recipients and critically ill recipients, with a sufficient volume or weight of the hemi-liver grafts, high-risk donors, long ischemia time (>10 hours), steatosis grafts, retransplantation patients, and low-volume LT centers were the most mentioned prognostic factors.^[10,18,60–63]^ Of these factors, preoperative estimation of graft weight for recipient matching could be the most unpredictable factor, as it is usually a major concern affecting outcomes in adult SPLT. Although many equations were used to calculate liver volume, none of them were able to estimate the true liver volume and graft weight. Therefore, hemi-liver graft recovery from a deceased donor may be inadequate for matched adult recipients, leading to small-for-size grafts as well as graft failure.

## 5 Optimization of SPLT program

Organ shortage is the most important hindrance to organ transplantation worldwide. Although LDLT is increasingly performed to overcome the shortage of liver donations for transplantation, splitting the liver into 2 hemi-liver grafts could be another promising strategy to maximize organ donation from a deceased donor and increase the donor pool for LT. The answer to whether 1 plus 1 equals 2, in terms of SPLT, might be obvious and sadly “no.”^[64,65]^ However, splitting the liver graft can undoubtedly increase graft availability and potentially increase the number of adult transplant recipients. Although few reports have reported unsatisfactory results, SPLT should not be discouraged because of the considerably high incidence of patient mortality in the wait-list.

Moreover, improving allocation and organ sharing policies by the transplantation society might help encourage the widespread diffusion of SPLT for adult recipients. The split-liver network program, as proposed by the North Italy Transplant Organization, is a promising strategy for the implementation of SPLT.^[17]^ The SPLT network program can be established under the National Liver Transplantation Organization. Accordingly, transplantation centers with adequate experience in SPLT or willing to develop SPLT could join the program. Subsequently, these transplantation centers could closely cooperate in terms of organ sharing and surgical techniques, under the surveillance of the SPLT program.

Additionally, informed consent on the willingness to accept a split hemi-liver graft should be signed by patients when they are registered on the waiting list. All potential donors who meet the criteria for split-liver grafts should be included in the SPLT program for organ sharing. The SPLT program can make real-time matches of hemi-liver grafts with recipients who had signed the informed consent of SPLT, and on the basis of prioritization of national organ sharing with size-matched graft/recipients. Importantly, trust and collaboration between different centers are fundamental for successfully sharing hemi-liver grafts under this program as well.

## 6 Conclusion

As a result of improvements in surgical techniques and perioperative patient care, LT has now become a common and routine operation in many transplantation centers worldwide. Although the surgical technique may be a crucial factor for the success of LT, proper donor and recipient selection is of the utmost importance for the success of SPLT and affects long-term graft and patient survival. Meanwhile, more widespread use of SPLT for 2 adults would also improve outcomes by growing experience and refinement of surgical techniques. Furthermore, there is an urgent need to establish a formal organ-sharing program in terms of SPLT for pediatric, adult, or adult recipients under current organ transplantation organizations. Ultimately, the widespread diffusion of SPLT in the transplantation community may increase graft availability, benefit patients awaiting LT, and mitigate the high incidence of wait-list mortality in the setting of extreme organ shortage.

## Acknowledgments

This work was supported by grants from the Chang Gung Medical Research Program (CMRPG3K1461) to K.-M. Chan. In addition, the authors wish to thank Miss Ingrid Kuo and the Center for Big Data Analytics and Statistics (Grant no. CLRPG3D0048) at Chang Gung Memorial Hospital for creating the illustrations used herein.

## Author contributions

**Acquisition of data:** Kun-Ming Chan, Hao-Chien Hung, Jin-Chiao Lee, Tsung-Han Wu, Chih-Hsien Cheng, Chen-Fang Lee, Ting-Jung Wu, Hong-Shiue Chou, Wei-Chen Lee.

**Conceptualization:** Kun-Ming Chan, Wei-Chen Lee.

**Critical revision of the manuscript for important intellectual content:** Kun-Ming Chan, Wei-Chen Lee.

**Data curation:** Kun-Ming Chan, Hao-Chien Hung, Jin-Chiao Lee, Tsung-Han Wu, Yu-Chao Wang, Chih-Hsien Cheng, Chen-Fang Lee, Ting-Jung Wu, Hong-Shiue Chou.

**Investigation:** Kun-Ming Chan.

**Manuscript drafting:** Kun-Ming Chan.

**Supervision:** Wei-Chen Lee.

**Validation:** Kun-Ming Chan.

**Writing – original draft:** Kun-Ming Chan.
